# Enhanced nitrogen removal via simultaneous nitrification and denitrification by a newly isolated strain *Enterobacter cloacae* GW6 from estuarine sediment

**DOI:** 10.1371/journal.pone.0349379

**Published:** 2026-05-15

**Authors:** Shufeng Chen, Zhicai Zhang, Yinhua Wang, Chunping Wang, Xin Zhang, Xu Wang, Yalin Wu, Yidong Wang, Hongyu Guo

**Affiliations:** 1 The Department of Water Eco-Environment, Beijing Municipal Research Institute of Eco-Environmental Protection, Beijing, China; 2 Tianjin Key Laboratory of Animal and Plant Resistance, College of Life Sciences, Tianjin Normal University, Tianjin, China; 3 The State Key Laboratory of Estuarine and Coastal Research, East China Normal University, Shanghai, China; 4 Tianjin Key Laboratory of Water Resources and Environment, Tianjin Normal University, Tianjin, China; Universidade Catolica Portuguesa, PORTUGAL

## Abstract

Excessive nitrogen has been discharged into natural environments due to anthropogenic activities, leading to numerous negative impacts on natural ecosystems worldwide and becoming a global environmental issue. Therefore, effective removal of nitrogenous contaminants from natural ecosystems is crucial for the protection of both ecological sustainability and human interests. In this study, a strain of *Enterobacter cloacae* GW6 was newly isolated from natural estuarine sediment. This strain could utilize ammonium, nitrite and nitrate, with removal efficiencies of 99.60%, 93.73% and 98.79%, and the maximum removal rates of 39.96, 40.39 and 44.65 mg/L/h, respectively. Importantly, Strain GW6 showed excellent simultaneous heterotrophic nitrification and aerobic denitrification (HN-AD) capability with the maximum TN removal rate of 35.27 mg/L/h, which is greater than those of many other HN-AD bacteria reported previously, indicating that Strain GW6 was suitable for the efficient nitrogen removal in aquatic ecosystems with high ammonium, nitrite and nitrate concentrations. The detection of functional genes *amoA*, *hao*, *napA*, *nirK* and *nosZ* from Strain GW6 further confirmed its HN-AD capability. Strain GW6 exhibited strong nitrogen removal capability across a wide range of environmental conditions, which makes it a promising candidate as a HN-AD bacterium for the effective biological treatment of nitrogen contamination and eutrophication in natural aquatic ecosystems, thereby contributing to better environmental protection.

## 1. Introduction

In recent years, excessive nitrogen has been discharged into natural environments due to anthropogenic activities, leading to numerous negative impacts on natural ecosystems worldwide and becoming a global ecological and environmental issue [1]. Increasing nitrogen input in aquatic ecosystems could result in eutrophication, which would further cause water quality deterioration, and thus threaten ecological sustainability and human health [2]. Therefore, effective removal of nitrogenous contaminants from environments is crucial for the protection of both ecological sustainability and human interests.

Biological approaches for nitrogen removal are superior to traditional physical and chemical treatment techniques, because they have high efficiency and relatively low cost, and they are more environmentally friendly [3]. Whereas, the conventional processes of biological nitrogen removal need two steps, i.e., firstly autotrophic nitrifiers perform the aerobic nitrification process, and then heterotrophic denitrifiers perform the anaerobic denitrification process, and thus are relatively complex [4].

In the 1980s, a species of bacteria, *Thiosphaera pantotropha*, that could perform heterotrophic nitrification and aerobic denitrification (HN-AD) simultaneously was discovered [5], which provided a unique way to treat nitrogenous contaminants efficiently. Since then, many HN-AD bacteria belonging to a broad range of genera have been reported, such as *Vibrio diabolicus* SF16 [6], *Pseudomonas tolaasii* Y-11 [7], *Ochrobactrum anthropic* LJ81 [8], *Acinetobacter tandoii* MZ-5 [9], *Acinetobacter* sp. ND7 [3], *Pseudomonas mendocina* X49 [10], *Ochrobactrum anthropi* HND19 [11].

However, to date, most of the reported HN-AD bacteria have been isolated from man-made systems, such as, municipal activated sludge, landfill leachate, wastewater treatment plants, laboratory reactors, etc. [12]. However, the conditions in these man-made systems are very different from those in natural environments, and thus, the HN-AD bacteria isolated from these man-made systems may not grow well in natural environments, and therefore may not be truly suitable for effectively treating nitrogen contamination in natural ecosystems [9]. In contrast, strains derived from natural environments, particularly estuarine systems, often demonstrate greater tolerance to environmental fluctuations than those enriched from acclimated activated sludge. Therefore, exploring HN-AD bacteria derived from natural ecosystems is critically important for the effective treatment of nitrogen contamination in natural ecosystems.

Moreover, it has been observed that nitrite accumulation would occur when some HN-AD bacteria remove ammonium and nitrate simultaneously, which would decrease the removal efficiency of total nitrogen [13]. Nitrite accumulation could also be toxic to environmental microorganisms [14]. Whereas, for HN-AD bacteria, the nitrite removal ability has been rarely examined [3,8,9,15]. Therefore, exploring HN-AD bacteria derived from natural environments with strong nitrite removal capabilities is also crucial for better management of nitrogen contamination in natural ecosystems.

In this study, a naturally derived strain, GW6, with HN-AD capability, was isolated from the sediment of the Yongdingxin River estuary area, China. This strain was identified as *Enterobacter cloacae* GW6, based on its phenotypic and phylogenetic characteristics. The nitrogen removal performance of Strain GW6 was explored with different nitrogen sources. Moreover, the HN-AD capability of Strain GW6 under different conditions of carbon source, C/N ratio, salinity, pH, temperature, rotation speed, and initial nitrogen concentration was examined. In addition, the functional genes in the nitrogen transformation processes by Strain GW6 were investigated, and the possible pathway of heterotrophic nitrification and aerobic denitrification was proposed. This study may facilitate the application of biological nitrogen removal in natural aquatic ecosystems, thereby contributing to environmental protection by providing insights into the simultaneous HN-AD characteristics of the naturally derived strain *E. cloacae* GW6.

## 2. Materials and methods

### 2.1. Medium

The enrichment medium (EM) [3] per liter comprised KNO_3_ 1.5g, KH_2_PO_4_ 1.5g, MgSO_4_·7H_2_O 0.2g, and FeSO_4_ 0.01g, with sucrose 10g for bacteria enrichment. Heterotrophic nitrification medium (HNM) [16] per liter comprised K_2_HPO_4_ 7.0g, KH_2_PO_4_ 3.0g, MgSO_4_·7H_2_O 0.1g, FeSO_4_·7H_2_O 0.05g and (NH_4_)_2_SO_4_ 1.89g, with sucrose 10g. Denitrification medium (DM) [7] per litter comprised K_2_HPO_4_ 7.0g, KH_2_PO_4_ 3.0g, MgSO_4_·7H_2_O 0.1g, FeSO_4_·7H_2_O 0.05g, NaNO_2_ 1.97g (DM-1) or KNO_3_ 2.89g (DM-2), with sucrose 10g. Simultaneous nitrification and denitrification medium (SNDM) [7] per litter comprised K_2_HPO_4_ 7.0g, KH_2_PO_4_ 3.0g, MgSO_4_·7H_2_O 0.1g, FeSO_4_·7H_2_O 0.05g, (NH_4_)_2_SO_4_ 0.943g and KNO_3_ 1.44g, with sucrose 10g. The Luria-Bertani medium (LB), which per litter comprised tryptone 10g, yeast extract 5g, and NaCl 10g, was used for culture preservation [3]. For all the mediums, the initial pH was adjusted to 7.0, and all the media were sterilized with an autoclave for 30 min at 121℃ before use.

### 2.2. Isolation and identification

Sediment samples from the Yongdingxin River estuary area (39º05′ N, 117º46′ E; no permits were required to access this area), China were collected. For each sediment sample, a fresh sample of 10g was incubated in a flask (500 ml in size) with 200 ml EM at 150 rpm and 35℃. Every 12 h, additional nitrogen, and other nutrients were added into the incubation system. After cultivation for 48 h, through 10-fold serial dilution, the bacterial suspensions were streaked on SNDM agar plates and incubated at 35℃ for 3 days. Through repeated purification steps, eight HN-AD bacteria strains were isolated. Among these strains, an HN-AD bacteria strain (Strain GW6) with the strongest nitrogen removal capability was selected and stored for further investigation.

To identify Strain GW6, the genomic DNA of the strain was extracted and purified by using a bacterial genome DNA-extracting kit (Sangon Biotech, Shanghai, China), and the 16S rRNA gene was amplified by PCR. The PCR primers and protocol are shown in S1 Table. The sequencing of the amplified product was conducted by Sangon Biotech Co., Ltd. (Shanghai, China), and then we used the BLAST program from the NCBI (http://www.ncbi.nlm.nih.gov/blast/Blast.cgi) to compare the sequence. Then, we used MEGA 5.0 (neighbor-joining method) to construct a phylogenetic tree based on the results of sequence comparison.

### 2.3. Nitrogen removal performance of Strain GW6

To explicitly estimate the nitrogen removal performance of Strain GW6, the strain was cultivated at 35°C and 150 rpm in a flask (500 mL in size) with 100 mL LB medium for 16 h. We centrifuged the resulting bacterial suspension at 4000 rpm for 10 min at 4℃, and then removed the residual nitrogen source by washing the bacterial suspension three times with sterile 0.85% NaCl solution. The heterotrophic nitrification medium (HNM) was used to examine the heterotrophic nitrification capability of Strain GW6. The initial concentration of ammonium was 400 mg/L (a common initial nitrogen concentration in studies of bacterial HN-AD performance [7,17]). Denitrification medium (DM) was used to examine the aerobic denitrification capability of Strain GW6. The initial concentration of nitrite (DM-1) or nitrate (DM-2) was 400 mg/L, respectively. Simultaneous nitrification and denitrification medium (SNDM) was used to examine the simultaneous HN-AD capability of Strain GW6, with the initial ammonium of 200 mg/L and initial nitrate of 200 mg/L (resulting in an initial total nitrogen concentration of 400 mg/L). To examine the removal capability of organic nitrogen of Strain GW6, tryptone (2.86 g/L; containing 14% nitrogen, corresponding to an initial TN of 400 mg/L) was used in DM instead of other nitrogen sources [7].

We conducted all the experiments using flasks (250 mL) containing medium (100 mL), which were sterilized with an autoclave before use. The media were inoculated with bacterial suspension (2% inoculation size (v/v)) and cultivated for 24 h at 35°C and 150 rpm under aerobic conditions with dissolved oxygen level approximately 5 mg/L. Every 4 h, each medium was sampled and analyzed to determine bacterial growth (OD_600_), as well as ammonium, nitrite, nitrate and TN concentrations. The experiments were conducted with five replicates. To investigate whether the nitrogen removal by Strain GW6 was due to its active metabolism or other processes, control experiments were conducted with media lacking Strain GW6 inoculation, and with media inoculated with Strain GW6 but without nitrogen sources.

### 2.4. Effects of different environmental factors on HN-AD capability of Strain GW6

To examine the effects of different environmental factors on the HN-AD performance of Strain GW6, single-factor experiments (carbon source, C/N ratio, salinity, pH, temperature, rotation speed, and initial nitrogen concentration) were conducted using SNDM with initial ammonium of 200 mg/L and initial nitrate of 200 mg/L (initial TN of 400 mg/L), except for the initial nitrogen concentration experiment.

In the experiment for carbon source, sucrose, glucose, sodium citrate, sodium acetate, and potassium sodium tartrate were added to SNDM (C/N = 10), respectively. In the experiment for C/N ratio, the C/N ratio of SNDM (carbon source: sucrose) was set at 1, 5, 10, 15 and 20, respectively. In the experiment for salinity, the salinity of SNDM (carbon source: sucrose; C/N = 10) was adjusted to 0‰, 10‰, 20‰, 30‰ and 40‰, respectively. In the experiment for pH, the pH of SNDM (carbon source: sucrose; C/N = 10; salinity of 0‰) was set at 5, 6, 7, 8 and 9, respectively. All these experiments above were conducted at 35 ℃ and 150 rpm. In the experiment for temperature, cultivation temperature was set at 15°C, 20°C, 25°C, 30°C and 35°C, respectively (carbon source: sucrose; C/N = 10; salinity of 0‰; pH of 7; rotation speed of 150 rpm). In the experiment for rotation speed, the rotation speed was set at 100, 120, 150, 180 and 200 rpm, respectively (carbon source: sucrose; C/N = 10; salinity of 0‰; pH of 7; temperature of 35°C). In the experiment for initial nitrogen concentration, the initial nitrogen concentration of SNDM was adjusted to 100, 200, 400, 600 and 800 mg/L, respectively (with equal concentrations of ammonium and nitrate; carbon source: sucrose; C/N = 10; salinity of 0‰; pH of 7; temperature of 35°C; rotation speed of 150 rpm).

All these experiments were conducted using flasks (250 mL) with sterilized medium (100 mL, 2% inoculation size (v/v)). Control experiments were performed using media without Strain GW6 inoculation, and with media inoculated with Strain GW6 but devoid of nitrogen sources. All the experiments were conducted for 24 h, with five replicates. Every 4 h, each medium was sampled and analyzed to determine bacterial growth (OD_600_) and concentrations of ammonium, nitrite, nitrate and total nitrogen.

### 2.5. Investigation of functional genes using PCR

The genome DNA of Strain GW6 was extracted and purified by using the bacterial genome DNA-extracting kit (Sangon Biotech, Shanghai, China). Then the genome DNA was used as the template for the key functional genes involved in the processes of nitrification and denitrification, including: *amoA*, *hao*, *napA*, *nirK*, and *nosZ*. The PCR primers and protocols are shown in S1 Table.

### 2.6. Analytical methods

The nitrogen concentration of ammonia was measured using the indophenols blue spectrophotometry method [7]. The nitrogen concentration of nitrate was determined by subtracting two times of the background absorbance value at 275 nm from the absorbance value at 220 nm [7]. The nitrogen concentration of nitrite was measured using the N-(1-naphthyl)-1,2-diaminoethane spectrophotometry method [3]. Total nitrogen concentration was determined by subtracting two times of the background absorbance value at 275 nm from the absorbance value at 220 nm after using alkaline potassium persulfate digestion [7].

The removal efficiencies (REs) of ammonium, nitrite, nitrate and TN were calculated using the following equation: RE (%) = (C_0_-C_f_) / C_0_ × 100%, where C_0_ and C_f_ are the initial and final (after cultivation for 24 h) concentrations of ammonium, nitrite, nitrate or TN, respectively. One-way ANOVAs and Tukey HSD tests (significance level of *P* < 0.05) were conducted using SPSS Statistics 21.0 software to examine the environmental factor effects on the removal efficiencies for ammonium, nitrite, nitrate and TN.

## 3. Results and discussion

### 3.1. Strain GW6 identification

In the preliminary study, a total of eight strains with HN-AD capability were isolated. Among these, Strain GW6 demonstrated the highest nitrogen removal performance, removing approximately 99% of ammonium and 98% of nitrate within 24 hours, while the other strains removed around 90–95% of ammonium and nitrate within the same time frame. Based on this superior nitrogen removal efficiency, Strain GW6 was selected as the candidate strain for further investigation. The results from our preliminary study on Strain GW6 showed that in the medium without an organic carbon source, the growth of Strain GW6 was inhibited. Moreover, under anaerobic conditions, we observed no nitrogen removal by Strain GW6. Thus, Strain GW6 had heterotrophic and aerobic characteristics.

The control experiments demonstrated that nitrogen removal by Strain GW6 was attributed to its active metabolism rather than other processes. In the absence of nitrogen sources, there was no significant nitrogen removal observed when Strain GW6 was inoculated. Similarly, when Strain GW6 was not inoculated into the medium, no nitrogen removal occurred. These findings confirm that the observed nitrogen removal in the presence of nitrogen sources was primarily due to the active metabolic processes of Strain GW6, such as heterotrophic nitrification and aerobic denitrification.

On LB medium, the appearance of Strain GW6 colony was circular, off-white, semitransparent, and slightly bulged with regular edges and smooth surfaces. Strain GW6 had a similarity of 99.56% with *Enterobacter cloacae* strain *ATCC* 13047, based on the BLAST results. The phylogenetic tree also revealed that Strain GW6 belonged to the genus *Enterobacter* (S2 Figure). We submitted the nucleotide sequence of Strain GW6 to the GenBank database (Accession Number: OR244057).

### 3.2. Heterotrophic nitrification performance of Strain GW6

**Fig 1:**
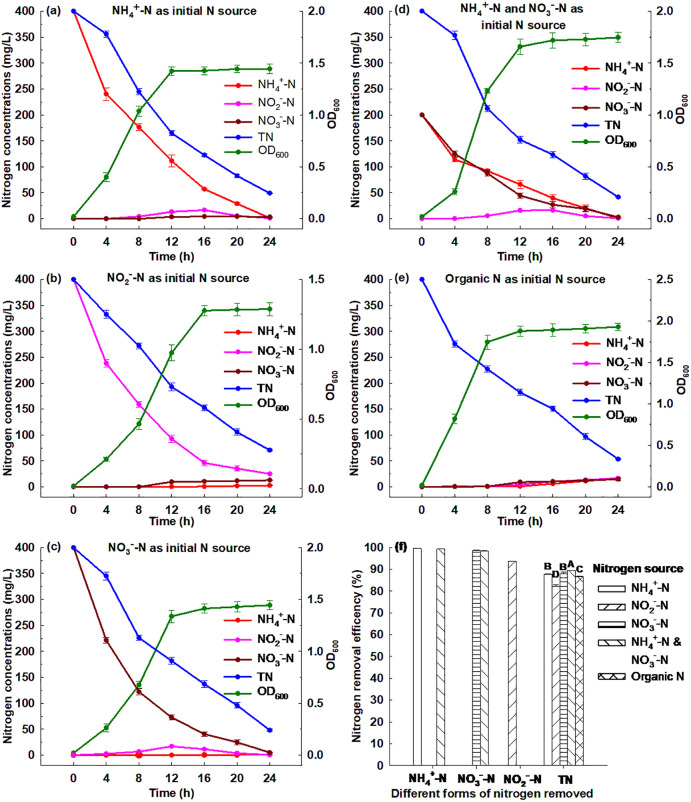
Simultaneous heterotrophic nitrification and aerobic denitrification performances of *Enterobacter cloacae* GW6 with (a) NH_4_^+^-N, (b) NO_2_^-^-N, (c) NO_3_^-^-N, (d) NH_4_^+^-N and NO_3_^-^-N (equal initial concentrations), (e) organic N as the initial N sources, respectively, and (f) the corresponding nitrogen removal efficiencies for different forms of nitrogen removed. Data are means ± SE (n = 5). For TN removal efficiency in the panel f, different letters indicate significant difference (Tukey HSD test, *P* < 0.05).

Figure 1a shows that Strain GW6 grew quickly in the first 12 h (a logarithmic phase), and then remained relatively stable status (a stationary phase) from 12 to 24 h. Within 24 h cultivation, the ammonium concentration decreased from 400 to 1.60 mg/L and Strain GW6 grew substantially with the OD_600_ increasing from 0.02 to 1.44. These suggested that the ammonium removal and Strain GW6 growth were closely related. The maximum ammonium removal rate of 39.96 mg/L/h was achieved within the first 4 h, which was higher than that of many other nitrifying bacteria reported previously: *Alcaligenes aquatilis* AS1 (maximum 30.5 mg/L/h), *Alcaligenes faecalis* No. 4 (28.9 mg/L/h), and *Pseudomonas mendocina* X49 (26.39 mg/L/h) [10,18,19]. Within 24 h, Strain GW6 removed 99.60% of ammonium (Fig 1f), and the average ammonium removal rate was 16.60 mg/L/h, which was higher than that of many nitrifying bacteria reported previously: *Cupriavidus* sp. S1 (10.43 mg/L/h), *Pseudomonas mendocina* X49 (10.00 mg/L/h), and *Pseudomonas stutzeri* YG-24 (8.75 mg/L/h) [10,20,21].

In the nitrification process by Strain GW6, the nitrite and nitrate concentrations increased slightly and then decreased to very low levels. It has been suggested that nitrite and nitrate would appear in the nitrification process as the main intermediate products [22]. Both nitrite and nitrate could be consumed by the denitrification by Strain GW6, which was consistent with other HN-AD bacteria reported previously: *Acinetobacter* sp. HA2, *Diaphorobacter polyhydroxybutyrativorans* SL-205, and *Ochrobactrum anthropic* LJ81 [8,23,24]. Thus, Strain GW6 exhibited strong heterotrophic nitrification performance.

### 3.3. Aerobic denitrification performance of Strain GW6

Figure 1b shows that in DM-1, Strain GW6 grew rapidly (a logarithmic phase) within 16 h, and then remained relatively stable status (a stationary phase) from 16 to 24 h. The growth of Strain GW6 was also associated with the nitrite removal, as the nitrite concentration decreased from 400 to 25.08 mg/L and Strain GW6 grew substantially with the OD_600_ increasing from 0.02 to 1.29 within 24 h cultivation. During the cultivation process, a low concentration of nitrate was detected. Such phenomena were also found for other denitrifying bacteria reported previously: *Diaphorobacter polyhydroxybutyrativorans* SL-205 and *Ochrobactrum anthropic* LJ81 [8,24]. The highest removal rate for nitrite reached 40.39 mg/L/h within the first 4 h, which was greater than many other bacteria reported previously: *Enterobacter cloacae* CF-S27 (11.2 mg/L/h), *Pannonibacter* sp. W30 (10.77 mg/L/h) and *Acinetobacter junii* YB (8.45 mg/L/h) [15,25,26]. Within 24 h, 93.73% of nitrite was removed by Strain GW6 (Fig 1f), with an average removal rate of 15.62 mg/L/h, which was higher than many other denitrifying bacteria reported previously: *Cupriavidus* sp. S1 (8.36 mg/L/h), *Pseudomonas stutzeri* YG-24 (7.51 mg/L/h) and *Pseudomonas mendocina* X49 (4.54 mg/L/h) [10,20,21]. These suggest that Strain GW6 could perform aerobic denitrification effectively.

Figure 1c shows that in DM-2, Strain GW6 grew quickly (a logarithmic phase) in the first 12 h, then grew slowly between 12−16 h, and reached the stationary phase between 16 and 24 h. The nitrate removal and Strain GW6 growth were closely related, as the nitrate concentration decreased from 400 to 4.85 mg/L and Strain GW6 grew substantially with OD_600_ increasing from 0.02 to 1.45 within 24 h cultivation. During the cultivation of Strain GW6 in DM-2, the nitrite concentration increased slightly and then decreased to very low levels. Similar phenomena were also observed for other denitrifying bacteria reported previously: *Pseudomonas stutzeri* D6, *Diaphorobacter polyhydroxybutyrativorans* SL-205 and *Acinetobacter* sp. ND7 [3,24,27]. The highest rate for nitrate removal reached 44.65 mg/L/h within the first 4 h, which was greater than many other bacteria reported previously: *Paracoccus versutus* LYM (33 mg/L/h), *Pseudomonas balearica* RAD-17 (21.7 mg/L/h) and *Acinetobacter junii* YB (7.98 mg/L/h) [15,28,29]. Within 24 h, Strain GW6 removed 98.79% of nitrate (Fig 1f), and the average rate for nitrate removal was 16.46 mg/L/h. This average nitrate removal rate was greater than many other bacteria reported previously: *Pseudomonas stutzeri* T13 (11.95 mg/L/h), *Zobellella taiwanensis* DN-7 (9.60 mg/L/h) and *Cupriavidus* sp. S1 (8.64 mg/L/h) [21,30,31].

### 3.4. Simultaneous HN-AD performance of Strain GW6

Figure 1d illustrates that in the SNDM, Strain GW6 grew quickly (a logarithmic phase) within the first 12 h, and then remained relatively stable status (a stationary phase) from 12 to 24 h. Within 24 h cultivation, Strain GW6 grew substantially with OD_600_ increasing from 0.02 to 1.75, meanwhile, the ammonium, nitrate and total nitrogen concentrations decreased from 200 to 1.16 mg/L, from 200 to 3.06 mg/L, and from 400 to 41.76 mg/L, respectively. This suggested that the growth of Strain GW6 was closely associated with the ammonium, nitrate and total nitrogen removal in the SNDM.

Within 24 h, 99.42% of ammonium, 98.47% of nitrate, and 89.56% of TN were removed by Strain GW6 (Fig 1f). In the SNDM, the average ammonium and nitrate removal rates by Strain GW6 were 8.28 and 8.21 mg/L/h, respectively. Although, these removal rates were lower than those in the HNM or DM-2, respectively, they remained at relatively high levels compared with those of the nitrogen removal bacteria reported previously (see discussions above). The highest removal rate for TN removal reached 35.27 mg/L/h within 8 h. Within 24 h, the average TN removal rate by Strain GW6 in the SNDM was 14.93 mg/L/h, which, although slightly lower than that of *Paracoccus denitrificans* HY-1 (15.33 mg/L/h) [32], was much higher than many other previously reported HN-AD bacteria, including, *Acinetobacter* sp. ND7 (2.34 mg/L/h) [3], *Pseudomonas tolaasii* Y-11 (0.17 mg/L/h) [7], *Acinetobacter tandoii* MZ-5 (2.88 mg/L/h) [9], *Pseudomonas aeruginosa* WS-03 (5.12 mg/L/h) [33], and *Acinetobacter* sp. G11 (6.59 mg/L/h) [34], etc.

The highest OD_600_ reached 1.75 when Strain GW6 was cultivated in the SNDM, which was higher than the maximum OD_600_ in the HNM (1.44), DM-1 (1.29), and DM-2 (1.45). These results showed that Strain GW6 could simultaneously and effectively utilize both ammonium and nitrate, and the mix of ammonium and nitrate could also further promote the growth of Strain GW6, which was consistent with the previous findings [3,8].

Although nitrite accumulation is commonly observed in HN-AD processes [13], the results in Fig 1d show that nitrite levels remained nearly undetectable throughout the study. This finding is significant as it suggests that Strain GW6 may possess efficient mechanisms to prevent nitrite accumulation, such as rapid nitrite reduction or a robust electron transfer system, which could not only limit nitrite buildup but also potentially enhance the overall efficiency of nitrogen removal in HN-AD processes. This positive outcome warrants further investigation, as it may provide valuable insights into enhancing the performance and stability of such processes in practical applications. These results illustrated that Strain GW6 has excellent simultaneous HN-AD capability, suggesting that Strain GW6 was suitable for the efficient nitrogen removal treatment in environments with high ammonium, nitrite and nitrate concentrations.

### 3.5. Nitrogen removal capability of Strain GW6 with an organic nitrogen source

In natural ecosystems, organic nitrogen is an important form of nitrogen [35], and a high concentration of organic nitrogen could lead to eutrophication by promoting algae growth in aquatic ecosystems [36,37]. Figure 1e shows that when organic nitrogen (tryptone) was supplied as the nitrogen source, Strain GW6 grew quickly (a logarithmic phase) within 8 h, then remained relatively stable (a stationary phase) from 8 to 24 h. Within 24 h cultivation, Strain GW6 grew substantially with the OD_600_ increasing from 0.02 to 1.93, meanwhile, the concentration of TN reduced from 400 to 53.45 mg/L. There was little ammonium, nitrite, and nitrate accumulation during the cultivation, which was consistent with HN-AD bacteria *Pseudomonas tolaasii* Y-11 [7]. After 24 h, 86.64% of TN was removed (Fig 1f), and the average removal rate for TN reached 14.44 mg/L/h, which was greater than that of *Pseudomonas tolaasii* Y-11 (0.25 mg/L/h) [7]. These results indicated that Strain GW6 was also suitable for organic nitrogen removal in natural ecosystems with eutrophication issues.

### 3.6. Effects of different environmental factors on Strain GW6 HN-AD performance

#### 3.6.1. Effect of carbon source.

**Fig 2:**
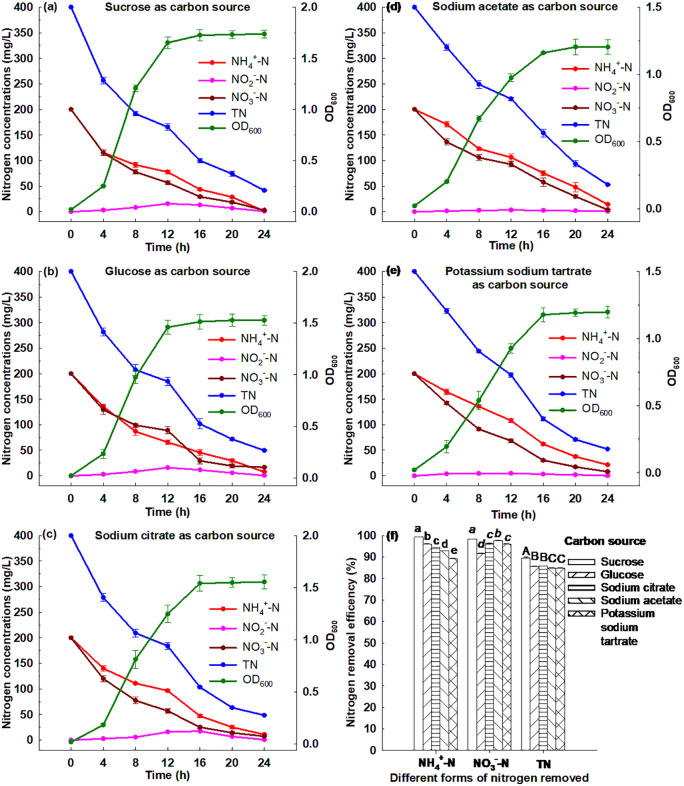
Simultaneous heterotrophic nitrification and aerobic denitrification performances of *Enterobacter cloacae* GW6 with ammonium-nitrogen and nitrate-nitrogen as the nitrogen sources under different carbon sources. Data are means ± SE (n = 5). In panel f, different letters within each form of nitrogen indicate significant differences (Tukey HSD test, *P* < 0.05).

Figure 2 shows that Strain GW6 grew well in the medium with the five carbon sources, respectively, and the OD_600_ reached between 1.20 and 1.74 after 24 h cultivation. Strain GW6 grew best in the medium with sucrose as the carbon source, and within 24 h, 99.42% of ammonium, 98.47% of nitrate and 89.61% of TN were removed (S3 Table). Strain GW6 grew worst in the medium with potassium sodium tartrate as the carbon source, and within 24 h, 89.28% of ammonium, 95.94% of nitrate and 86.91% of TN were removed (S3 Table). The results suggested that various carbon sources could be utilized by Strain GW6, and this strain would achieve the highest nitrogen removal efficiencies with sucrose as the carbon source, which was consistent with the previous findings [6,31,38].

#### 3.6.2. Effect of C/N ratio.

**Fig 3:**
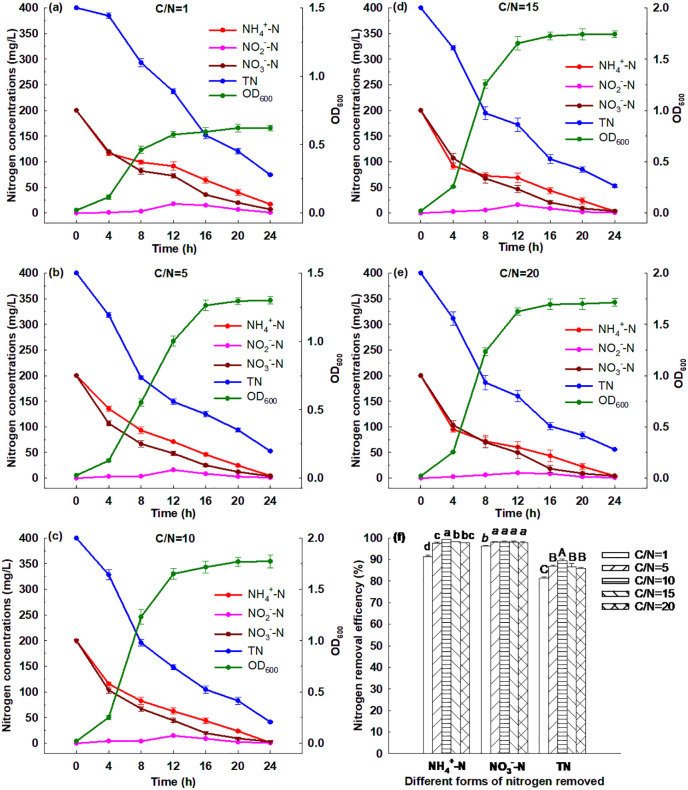
Simultaneous heterotrophic nitrification and aerobic denitrification performances of *Enterobacter cloacae* GW6 with ammonium-nitrogen and nitrate-nitrogen as the nitrogen sources under different C/N ratios. Data are means ± SE (n = 5). In panel f, different letters within each form of nitrogen indicate significant differences (Tukey HSD test, *P* < 0.05).

Figure 3 illustrates that Strain GW6 could grow in the media with different C/N ratios ranging from 1 to 20, and the OD_600_ reached between 0.62 and 1.77 after 24 h cultivation. Strain GW6 grew best in the medium with the C/N ratio of 10, and within 24 h, 99.41% of ammonium, 98.47% of nitrate and 89.64% of TN were removed (S3 Table). The enhanced nitrogen removal performance at this optimal C/N ratio could be attributed to the synergistic effects of both assimilation and dissimilatory processes. The assimilation process is crucial for incorporating inorganic nitrogen into organic biomass, thereby promoting the growth of Strain GW6 and enhancing overall nitrogen uptake. In contrast, dissimilatory processes, particularly denitrification, are vital for converting nitrate and nitrite into nitrogen gas, effectively eliminating nitrogen from the system. Our findings suggest that while denitrification plays a significant role in nitrogen removal, the importance of nitrogen assimilation should not be underestimated. This dual mechanism underscores the complexity of nitrogen cycling within ecosystems and highlights the necessity of optimizing both processes for effective nitrogen management. Although Strain GW6 showed the lowest growth in the medium with a C/N ratio of 1, it still removed 91.44% of ammonium, 96.39% of nitrate, and 81.38% of total nitrogen (TN) within 24 hours (S3 Table). These results suggested that a wide range of C/N ratio (1–20) could be adapted by Strain GW6, and the nitrogen removal efficiencies remained relatively high, which was superior to many other HN-AD bacteria reported previously: *Pseudomonas stutzeri* YG-24 (suitable C/N ratio: 2–10), *Vibrio diabolicus* SF16 (suitable C/N ratio: 4–14), *Acinetobacter tandoii* MZ-5 (suitable C/N ratio: 11–18) [6,9,20].

#### 3.6.3. Effect of salinity.

**Fig 4:**
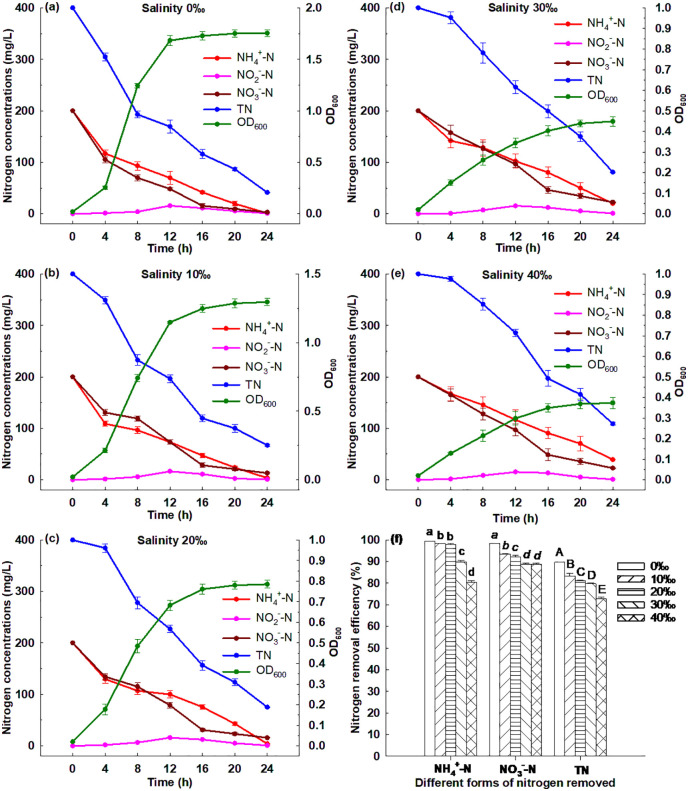
Simultaneous heterotrophic nitrification and aerobic denitrification performances of *Enterobacter cloacae* GW6 with ammonium-nitrogen and nitrate-nitrogen as the nitrogen sources under different salinities. Data are means ± SE (n = 5). In panel f, different letters within each form of nitrogen indicate significant differences (Tukey HSD test, *P* < 0.05).

Figure 4 illustrates that Strain GW6 could grow under different salinity ranging from 0‰ to 40‰, and the OD_600_ reached between 0.37 to 1.75 after 24 h cultivation. Strain GW6 grew best under salinity of 0‰, and within 24 h, 99.42% of ammonium, 98.47% of nitrate and 89.69% of TN were removed (S3 Table). Strain GW6 grew worst under salinity of 40‰, and within 24 h, 80.48% of ammonium, 88.75% of nitrate and 72.76% of TN were removed (S3 Table). This probably was due to the negative effects of high salinity on enzyme activities responsible for nitrogen removal [6]. The results suggested that a relatively wide range of salinity (0‰ to 40‰) could be adapted by Strain GW6, and this strain could still perform fairly efficient nitrogen removal, which was superior to many other HN-AD bacteria reported previously: *Aeromonas* sp. HN-02 (suitable salinity: 0‰-20‰), *Serratia marcescens* W5 (suitable salinity: 0‰-25‰), *Acinetobacter* sp. JR1 (suitable salinity: 0‰-20‰) [38–40].

The broad salinity adaptability of Strain GW6 could be attributed to several physiological and genetic mechanisms. This strain may employ osmoprotective strategies, including the synthesis of compatible solutes such as trehalose and betaine, which help stabilize proteins and cellular structures under osmotic stress [41]. Additionally, genes related to ion transport, such as Na ⁺ /H⁺ antiporters and K⁺ channels, may facilitate the regulation of intracellular ion concentrations, thereby maintaining an optimal Na ⁺ /K⁺ ratio under salt stress [42]. These adaptations not only highlight the ecological significance of Strain GW6 in estuarine environments, but also underscore its potential for bioremediation applications in saline regions.

#### 3.6.4. Effect of initial pH.

**Fig 5:**
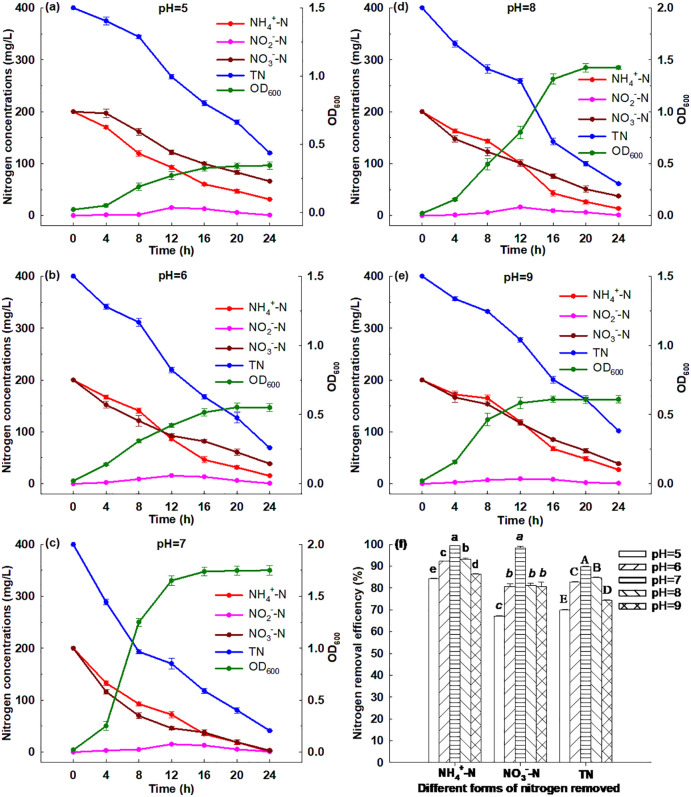
Simultaneous heterotrophic nitrification and aerobic denitrification performances of *Enterobacter cloacae* GW6 with ammonium-nitrogen and nitrate-nitrogen as the nitrogen sources under different initial pH. Data are means ± SE (n = 5). In panel f, different letters within each form of nitrogen indicate significant differences (Tukey HSD test, *P* < 0.05).

Figure 5 illustrates that Strain GW6 could grow under different initial pH values ranging from 5 to 9, and the OD_600_ reached between 0.34 and 1.75 after 24 h cultivation. Strain GW6 grew best under initial pH = 7, and within 24 h, 99.42% of ammonium, 98.48% of nitrate and 89.70% of TN were removed (S3 Table). Strain GW6 had decreased growth under an acidic or alkaline condition. Under the acidic condition of initial pH = 5, Strain GW6 had relatively low removal efficiencies of ammonium, nitrate and TN at 84.47%, 67.06% and 69.88%, respectively, after 24 h cultivation (S3 Table). Under the alkaline condition of initial pH = 9, Strain GW6 also had relatively low removal efficiencies of ammonium, nitrate and TN at 86.48%, 80.63% and 74.53%, respectively, after 24 h cultivation (S3 Table). This probably was due to the negative effects of extreme pH on bacterial metabolism involved in nitrogen removal [43]. The results suggested that a relatively wide range of pH (5–9) could be adapted by Strain GW6, and this strain still has fairly strong nitrogen removal capability, which was superior to many other HN-AD bacteria reported previously: *Bacillus methylotrophicus* L7 (suitable pH: 7–8), *Vibrio diabolicus* SF16 (suitable pH: 7.5–9.5), *Photobacterium* sp. NNA4 (suitable pH: 7–9) [6,44,45].

#### 3.6.5. Effect of temperature.

**Fig 6:**
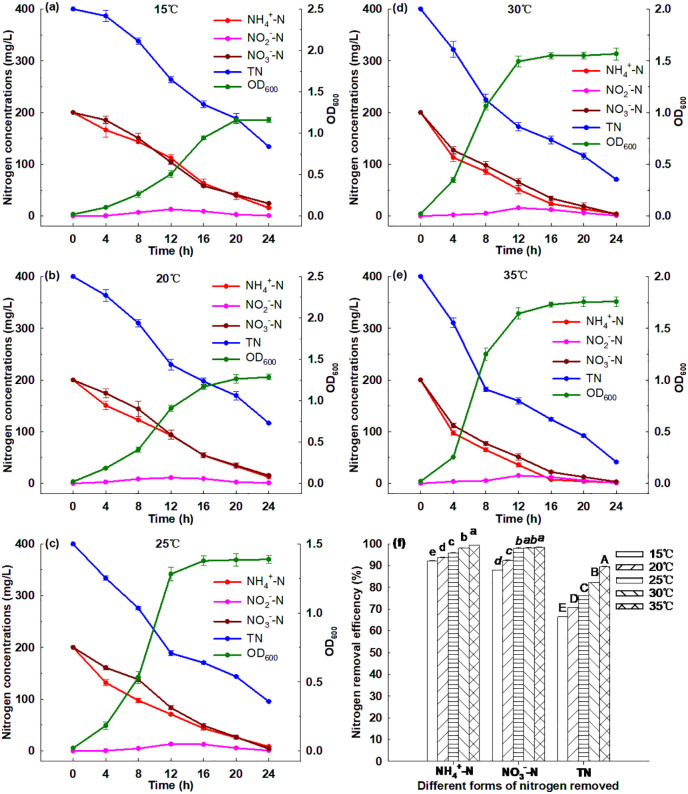
Simultaneous heterotrophic nitrification and aerobic denitrification performances of *Enterobacter cloacae* GW6 with ammonium-nitrogen and nitrate-nitrogen as the nitrogen sources under different temperatures. Data are means ± SE (n = 5). In panel f, different letters within each form of nitrogen indicate significant differences (Tukey HSD test, *P* < 0.05).

The HN-AD performance of Strain GW6 under different temperatures is illustrated in Fig 6. Strain GW6 could grow under different temperatures ranging from 15℃ to 35℃, and the OD_600_ reached between 1.16 to 1.76 after 24 h cultivation. Strain GW6 grew best under 35℃, and within 24 h, 99.41% of ammonium, 98.48% of nitrate and 89.68% of TN were removed (S3 Table). Strain GW6 grew worst under 15℃, and within 24 h, 92.07% of ammonium, 87.90% of nitrate and 66.50% of TN were removed (S3 Table). This probably was due to the negative effects of lower temperatures on the activities of nitrogen removal enzymes [3]. The results indicated that a relatively wide temperature range (15℃ to 35℃) could be adapted by Strain GW6, and this strain could maintain fairly high nitrogen removal efficiencies, which was superior to many other HN-AD bacteria reported previously: *Acinetobacter junii* YB (suitable temperature: 37℃), *Acinetobacter* sp. ND7 (suitable temperature:25℃-35℃), *Acinetobacter tandoii* MZ-5 (suitable temperature:25℃-30℃) [3,9,15].

#### 3.6.6. Effect of rotation speed.

**Fig 7:**
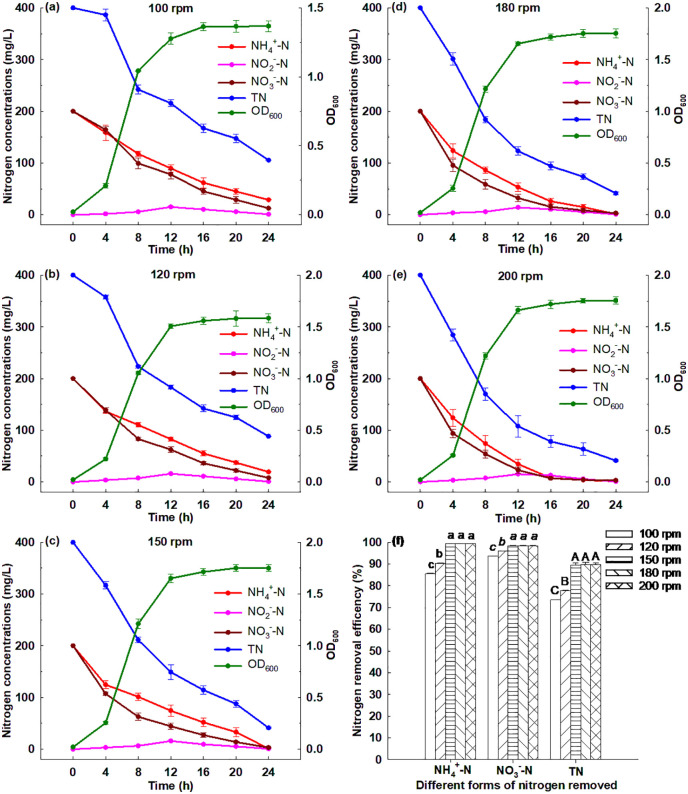
Simultaneous heterotrophic nitrification and aerobic denitrification performances of *Enterobacter cloacae* GW6 with ammonium-nitrogen and nitrate-nitrogen as the nitrogen sources under different rotation speeds. Data are means ± SE (n = 5). In panel f, different letters within each form of nitrogen indicate significant differences (Tukey HSD test, *P* < 0.05).

Figure 7 illustrates that Strain GW6 could grow well under different rotation speeds ranging from 100 to 200 rpm. As rotation speed increased, Strain GW6 exhibited both enhanced growth and HN-AD performance. Under rotation speed of 100 rpm, the OD_600_ of Strain GW6 was 1.37, and within 24 h, 85.58% of ammonium, 93.70% of nitrate and 73.64% of TN were removed (S3 Table). Under a rotation speed of 200 rpm, the OD_600_ of Strain GW6 was 1.75, and within 24 h, 99.43% of ammonium, 98.47% of nitrate and 89.71% of TN were removed (S3 Table). It has been suggested that dissolved oxygen plays a critical role in influencing bacterial growth and HN-AD performance [46,47]. Higher rotation speeds likely enhanced oxygen transfer efficiency and DO levels, which would facilitate the HN-AD performance of Strain GW6. Such a phenomenon was also observed for other HN-AD bacteria reported previously: *Arthrobacter arilaitensis* Y-10, *Acinetobacter* sp. JR1, *Ochrobactrum anthropic* LJ81 [8,39,48].

#### 3.6.7. Effect of initial nitrogen concentration.

**Fig 8:**
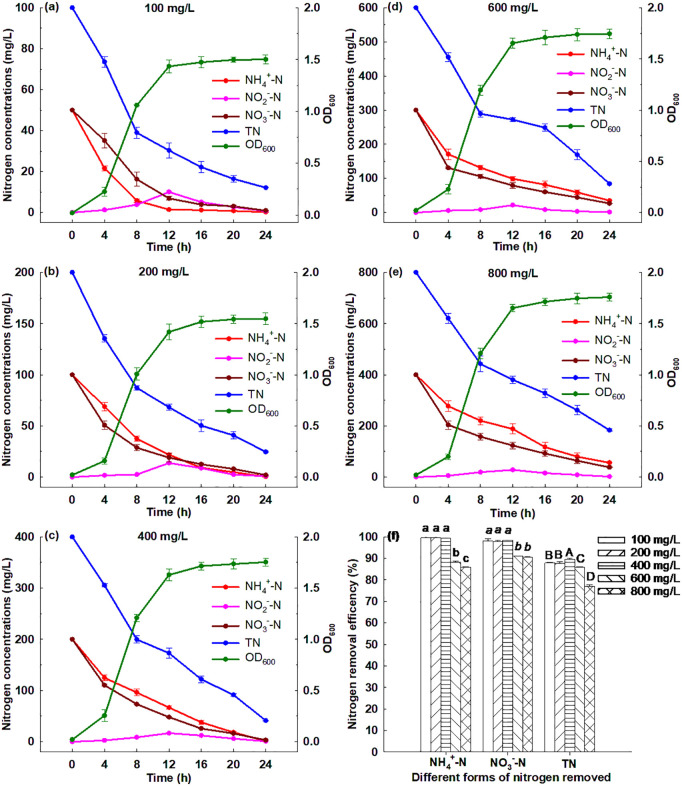
Simultaneous heterotrophic nitrification and aerobic denitrification performances of *Enterobacter cloacae* GW6 with ammonium-nitrogen and nitrate-nitrogen as the nitrogen sources under different initial nitrogen concentrations. Data are means ± SE (n = 5). In panel f, different letters within each form of nitrogen indicate significant differences (Tukey HSD test, *P* < 0.05).

Figure 8 illustrates that Strain GW6 grew well when the initial nitrogen concentration ranged from 100 to 800 mg/L, and the OD_600_ reached between 1.50 and 1.76 after 24 h cultivation. It could be observed that when the initial nitrogen concentration ranged from 100 to 400 mg/L, and after 24 h cultivation, ~ 99.55% of ammonium, ~ 98.18% of nitrate and ~88.42% of TN was removed (S3 Table); when the initial nitrogen concentration ranged from 600 to 800 mg/L, and after 24 h cultivation, ~ 87.16% of ammonium, ~ 90.81% of nitrate, and ~81.51% of TN was removed (S3 Table). These results showed that Strain GW6 has the capability to remove nitrogen effectively in a medium with a high initial nitrogen concentration, which was superior to many other HN-AD bacteria reported previously: *Acinetobacter* sp. ND7 (TN removal efficiency of 82.2% after 36 h, with initial TN concentration of 102.3 mg/L), *Aeromonas* sp. HN-02 (TN removal efficiency of 80.77% after 24 h, with initial TN concentration of 150 mg/L), *Pseudomonas tolaasii* Y-11 (TN removal efficiency of 20.6% after 72 h, with initial TN concentration of 419.17 mg/L) [3,7,40].

### 3.7. Nitrogen removal functional genes of Strain GW6

In general, the pathway of nitrogen conversion by HN-AD bacteria involves different functional enzymes [12]. The potential functional enzyme genes of Strain GW6 were amplified and analyzed. Fragments of the *amoA*, *hao*, *napA*, nir*K*, and *nosZ* genes were successfully amplified from Strain GW6 (S4 Figure).

The *amoA* gene encodes ammonia monooxygenase (AMO), a key enzyme in the first step of nitrification, converting ammonia (NH₃) to nitrite (NO₂⁻) [49]. In *E. cloacae* GW6, the amplification of *amoA* indicates that it possesses nitrification capability (S5 Figure). Moreover, *amoA* is involved in broader nitrogen cycling, interacting with genes responsible for denitrification and potentially impacting greenhouse gas emissions, such as nitrous oxide (N₂O). Overall, the *amoA* gene is essential for both nitrification and broader nitrogen transformations, with significant implications for environmental nitrogen management and bioremediation. The presence of *amoA* in *E. cloacae* GW6 aligns with its phenotype of rapid ammonium removal, consistent with previous reports [50,51], but contrasts with strains that rely solely on hydroxylamine oxidoreductase for heterotrophic nitrification.

The *hao* gene encodes hydroxylamine dehydrogenase (HAO), a critical enzyme in heterotrophic nitrification, where it catalyzes the conversion of hydroxylamine (NH₂OH) to nitrite (NO₂⁻) [52]. The detection of the *hao* gene confirms the heterotrophic nitrification capability of Strain GW6 (S5 Figure). The *hao* gene has been identified in other nitrifying bacteria such as *Acinetobacter junii* YB and *Thauera* sp. SND5 [15,53], highlighting its widespread role in nitrogen cycling and its contribution to nitrogen removal, thereby enhancing the overall efficiency of the nitrogen cycle.

The *napA* gene encodes nitrate reductase (NAP), an enzyme crucial for nitrate respiration and denitrification under aerobic conditions [54]. Its presence in Strain GW6 confirms the strain’s capability for aerobic denitrification (S5 Figure). The *napA* gene has also been detected in other HN-AD bacteria, such as *Acinetobacter* sp. ND7 and *Ochrobactrum anthropi* HND19 [3,11], indicating its widespread role in nitrogen cycling. By facilitating the reduction of nitrate to nitrite, NAP plays a key role in nitrogen removal processes, influencing the efficiency of denitrification in oxygen-rich environments.

The *nirK* gene encodes nitrite reductase (NIR), an enzyme that catalyzes the conversion of nitrite to nitric oxide during denitrification [12]. The detection of the *nirK* gene confirms the strain’s ability to reduce nitrite to nitric oxide (S5 Figure). The *nirK* gene is also found in other HN-AD bacteria, such as *Ochrobactrum anthropi* HND19 and *Acinetobacter* sp. TAC-1 [11,17], highlighting its role in the denitrification pathway. By facilitating this crucial step, *nirK* contributes to nitrogen removal and helps regulate nitrogen levels in ecosystems, influencing the overall efficiency of denitrification.

The *nosZ* gene encodes nitrous oxide reductase (N2OR), which mediates the final step of denitrification, converting nitrous oxide (N_2_O) to nitrogen gas (N_2_) [55]. The detection of *nosZ* in Strain GW6 indicates its potential to reduce N_2_O to environmentally benign N_2_ (S5 Figure). This gene has been found in a few other HN-AD bacteria, such as *Pseudomonas* sp. JQ-H3 and *Ochrobactrum anthropi* HND19 [11,50], emphasizing its role in mitigating greenhouse gas emissions. By facilitating the conversion of N_2_O, *nosZ* contributes to nitrogen removal and enhances the sustainability of nitrogen cycling in ecosystems, though further gas-phase studies are needed to evaluate its effectiveness in Strain GW6.

To further elucidate the genomic potential underlying the HN-AD capability of Strain GW6, it is essential to consider the electron flow and energy metabolism that couple these two processes. In *E. cloacae*, the denitrification pathway is intimately linked to the respiratory electron transport chain, and the membrane-bound nitrate reductase (Nar), encoded by the *nar* operon (*narG*, *narH*, and *narI*), transfers electrons from the quinone pool to nitrate, thereby contributing to proton motive force (PMF) generation and ATP synthesis [56]. Notably, *E. cloacae* exhibits respiratory flexibility through the expression of distinct membrane-bound reductases: nitrate reductase for denitrification and alternative reductases (e.g., selenate reductase) that can support PMF generation and sustain cell viability when nitrate is depleted [57]. During aerobic denitrification, oxygen and nitrate may serve as alternative terminal electron acceptors, with electron flux partitioned between the cytochrome bc_1_ complex and terminal reductases [58]. This modular electron transport chain, capable of routing electrons from carbon oxidation through denitrification pathways, may maximize energy yield under varying oxygen tensions and underpin the high nitrogen removal rates observed in Strain GW6.

## 4. Conclusions

This study demonstrated the excellent nitrogen removal capability of *E. cloacae* GW6, highlighting its potential for mitigating nitrogen pollution in aquatic ecosystems. In practical applications, strain GW6 could be a promising candidate for wastewater treatment and environmental bioremediation efforts, particularly in areas experiencing high ammonium, nitrite, and nitrate concentrations. To enhance its practical utility, future work should focus on optimizing the operational parameters for large-scale applications, including the strain’s performance under varying environmental conditions such as temperature, pH, and oxygen levels. Additionally, further research on the strain’s interactions with other microorganisms in complex ecological settings is needed to fully understand its behavior in natural environments. The development of bioreactors or biofilm systems could also improve its application in continuous nitrogen removal processes. Furthermore, to substantiate the proposed HN-AD mechanism, future studies will involve examining of the transcriptional expression of key functional genes under different cultivation conditions and time points. This will provide deeper insights into the functional roles of these genes and further enhance our understanding of strain GW6’s nitrogen removal pathways. By scaling up and fine-tuning the application of strain GW6, we can contribute to effective biological nitrogen removal and the reduction of eutrophication, advancing the protection of aquatic ecosystems worldwide.

## Supporting information

S1 Table PCR primers and protocols. (DOCX)

S2 Figure The neighbor-joining phylogenetic tree based on 16S rRNA gene sequences. *Enterobacter cloacae* GW6 is marked by the asterisk.
(DOCX)

S3 Table NH_4_^+^-N, NO_3_^-^-N and TN removal efficiencies under different carbon sources, C/N ratios, salinities, pH, temperatures, rotation speeds, and initial nitrogen concentrations by *Enterobacter cloacae* GW6 after 24 h cultivation. (DOCX)

S4 Figure The PCR amplification of *amoA*, *hao*, *napA*, *nirK* and *nosZ* functional genes from *Enterobacter cloacae* GW6. Markers: a, 10000 bp; b, 5000 bp; c, 3000 bp; d, 2000 bp; e, 1500 bp; f, 1000 bp; g, 750 bp; h, 500 bp; i, 250 bp; g, 100 bp.
(DOCX)

S5 Figure Proposed pathway of heterotrophic nitrification and aerobic denitrification of *Enterobacter cloacae* GW6 based on functional gene analysis using PCR. Arrows in purple color indicate the heterotrophic nitrification pathway, while arrows in orange color indicate the aerobic denitrification pathway.
(DOCX)
