# Supplementary material for: Enhanced nitrogen removal via simultaneous nitrification and denitrification by a newly isolated strain Enterobacter cloacae GW6 from estuarine sediment
Source: PLoS One. 2026 May 15;21(5):e0349379. doi: 10.1371/journal.pone.0349379 (PMC13178893; doi:10.1371/journal.pone.0349379)
Supplement: S1 Table — (DOCX) [file pone.0349379.s001.docx]

**S1 Table** PCR primers and protocols

| Gene | Primer sequence (5’-3’) and PCR protocol | Reference |
| --- | --- | --- |
| 16S rRNA | 27-F: AGAGTTTGATCCTGGCTCAG | Stanley *et al*. (1995) |
|  | 1492-R: TACGGTTACCTTGTTACGACTT |  |
|  | PCR protocol: Pre-denaturation at 94 °C for 5 min, and then 30 cycles of the following: denaturation at 94°C for 30 s, annealing at 55 °C for 30 s, and extension at 72 °C for 1 min. This was followed by a final extension at 72 °C for 10 min. |  |
| *amoA* | *amoA*-F: GGGAATTCAGAAATCCTGAAAGCGGC | Sinigalliano *et al*.(1995) |
|  | *amoA*-R: GGGGATCCGATACGAACGCAGAGAAG |  |
|  | PCR protocol: Pre-denaturation at 95 °C for 3 min, and then 30 cycles of the following: denaturation at 95 °C for 1 min, annealing at 50 °C for 30 s, and extension at 72 °C for 30 s. This was followed by a final extension at 72 °C for 10 min. |  |
| *hao* | *hao*-F: ATGTTTTGTGTNCAATGTGA | Padhi *et al*. (2017) |
|  | *hao*-R: GCYTTCAGYTCRAACCA |  |
|  | PCR protocol: Pre-denaturation at 94 °C for 5 min, and then 36 cycles of the following: denaturation at 94 °C for 1 min, annealing at 51.8 °C for 1min, and extension at 72 °C for 1 min. This was followed by a final extension at 72 °C for 10 min. |  |
| *napA* | *napA*-F: TCTGGACCATGGGCTTCAACCA | Kong *et al.* (2006) |
|  | *napA*-R: ACGACGACCGGCCAGCGCAG |  |
|  | PCR protocol: Pre-denaturation at 94 °C for 5 min, and then 35 cycles of the following: denaturation at 94 °C for 30 s, annealing at 59 °C for 30 s, and extension at 72 °C for 1 min. This was followed by a final extension at 72 °C for 10 min. |  |
| *nirK* | *nirK*-F: ATCATGGTSCTGCCGCG | Throback *et al.* (2004) |
|  | *nirK*-R: GCCTCGATCAGRTTGTGGTT |  |
|  | PCR protocol: Pre-denaturation at 94 °C for 2 min, and then 35 cycles of the following: denaturation at 94 °C for 30 s, annealing at 57 °C for 1 min, and extension at 72 °C for 1 min. This was followed by a final extension at 72 °C for 10 min. |  |
| *nosZ* | *nosZ*-F: CGYTGTTCMTCGACAGCCAG | Throback *et al.* (2004) |
|  | *nosZ*-R: CGCRASGGCAASAAGGTSCG |  |
|  | PCR protocol: Pre-denaturation at 94 °C for 2 min, and then 35 cycles of the following: denaturation at 94 °C for 30 s, annealing at 53 °C for 1 min, and extension at 72 °C for 1 min. This was followed by a final extension at 72 °C for 10 min. |  |

Note: The PCR mixture (50 μL) was composed of 1 μL DNA template, 2 μL F-primer, 2 μL R-primer, 5 μL 10×PCR buffer, 3 μL Mg^2+^ (25 mM), 2 μL dNTP (each 10 mM), 1 μL Taq DNA polymerase (5 U/μL), 34 μL dd H_2_O.

**References**

Kong, Q.-X., X.-W. Wang, M. Jin, Z.-Q. Shen, and J.-W. Li. 2006. Development and application of a novel and effective screening method for aerobic denitrifying bacteria. FEMS microbiology letters **260**:150-155.

Padhi, S. K., S. Tripathy, S. Mohanty, and N. K. Maiti. 2017. Aerobic and heterotrophic nitrogen removal by *Enterobacter cloacae* CF-S27 with efficient utilization of hydroxylamine. Bioresource Technology **232**:285-296.

Sinigalliano, C. D., D. N. Kuhn, and R. D. Jones. 1995. Amplification of the amoA gene from diverse species of ammonium-oxidizing bacteria and from an indigenous bacterial population from seawater. Applied and Environmental Microbiology **61**:2702-2706.

Stanley, J., N. Baquar, and A. Burnens. 1995. Molecular subtyping scheme for *Salmonella panama*. Journal of Clinical Microbiology **33**:1206-1211.

Throback, I. N., K. Enwall, A. Jarvis, and S. Hallin. 2004. Reassessing PCR primers targeting *nirS*, *nirK* and *nosZ* genes for community surveys of denitrifying bacteria with DGGE. Fems Microbiology Ecology **49**:401-417.
