# Supplementary material for: Enhanced nitrogen removal via simultaneous nitrification and denitrification by a newly isolated strain Enterobacter cloacae GW6 from estuarine sediment
Source: PLoS One. 2026 May 15;21(5):e0349379. doi: 10.1371/journal.pone.0349379 (PMC13178893; doi:10.1371/journal.pone.0349379)
Supplement: S2 Figure — Enterobacter cloacae GW6 is marked by the asterisk. (DOCX) [file pone.0349379.s002.docx]

**S2 Figure.** The neighbor-joining phylogenetic tree based on 16S rRNA gene sequences. *Enterobacter cloacae* GW6 is marked by the asterisk.
