# Supplementary material for: Enhanced nitrogen removal via simultaneous nitrification and denitrification by a newly isolated strain Enterobacter cloacae GW6 from estuarine sediment
Source: PLoS One. 2026 May 15;21(5):e0349379. doi: 10.1371/journal.pone.0349379 (PMC13178893; doi:10.1371/journal.pone.0349379)
Supplement: S3 Table — (DOCX) [file pone.0349379.s003.docx]

**S3 Table** NH_4_^+^-N, NO_3_^-^-N and TN removal efficiencies under different carbon sources, C/N ratios, salinities, pH, temperatures, rotation speeds, and initial nitrogen concentrations by *Enterobacter cloacae* GW6 after 24 h cultivation

| Factors | Level | Removal efficiency (%) | | |
| --- | --- | --- | --- | --- |
|  |  | NH_4_^+^-N | NO_3_^-^-N | TN |
| Carbon source | Sucrose | 99.42±0.02^a^ | 98.47±0.01^a^ | 89.61±0.13^a^ |
|  | Glucose | 96.18±0.05^b^ | 91.64±0.09^d^ | 87.57±0.07^b^ |
|  | Sodium citrate | 94.49±0.03^c^ | 96.22±0.11^c^ | 87.85±0.01^b^ |
|  | Sodium acetate | 92.92±0.03^d^ | 97.66±0.08^b^ | 86.80±0.10^c^ |
|  | Potassium sodium tartrate | 89.28±0.08^e^ | 95.94±0.12^c^ | 86.91±0.07^c^ |
| C/N ratio | 1 | 91.44±0.26^d^ | 96.39±0.07^b^ | 81.38±0.16^c^ |
|  | 5 | 97.68±0.15^c^ | 98.23±0.06^a^ | 86.84±0.18^b^ |
|  | 10 | 99.41±0.03^a^ | 98.47±0.08^a^ | 89.64±0.22^a^ |
|  | 15 | 98.34±0.03^b^ | 98.13±0.19^a^ | 86.75±0.45^b^ |
|  | 20 | 97.84±0.02^bc^ | 98.01±0.14^a^ | 86.04±0.10^b^ |
| Salinity | 0‰ | 99.42±0.01^a^ | 98.47±0.01^a^ | 89.69±0.02^a^ |
|  | 10‰ | 98.34±0.03^b^ | 93.55±0.01^b^ | 83.28±0.43^b^ |
|  | 20‰ | 98.00±0.12^b^ | 92.17±0.20^c^ | 81.21±0.04^c^ |
|  | 30‰ | 89.93±0.17^c^ | 88.75±0.13^d^ | 79.80±0.12^d^ |
|  | 40‰ | 80.48±0.25^d^ | 88.74±0.19^d^ | 72.76±0.32^e^ |
| pH | 5 | 84.47±0.05^e^ | 67.06±0.12^c^ | 69.88±0.10^e^ |
|  | 6 | 92.34±0.05^c^ | 80.76±0.41^b^ | 82.75±0.06^c^ |
|  | 7 | 99.42±0.01^a^ | 98.48±0.25^a^ | 89.70±0.12^a^ |
|  | 8 | 93.25±0.17^b^ | 81.29±0.30^b^ | 84.74±0.10^b^ |
|  | 9 | 86.48±0.10^d^ | 80.63±0.66^b^ | 74.53±0.03^d^ |
| Temperature | 15°C | 92.07±0.08^e^ | 87.90±0.05^d^ | 66.50±0.01^e^ |
|  | 20°C | 93.78±0.05^d^ | 92.39±0.07^c^ | 70.84±0.01^d^ |
|  | 25°C | 95.87±0.03^c^ | 97.89±0.15^b^ | 76.14±0.02^c^ |
|  | 30°C | 98.07±0.02^b^ | 98.24±0.07^ab^ | 82.35±0.01^b^ |
|  | 35°C | 99.41±0.04^a^ | 98.48±0.07^a^ | 89.68±0.02^a^ |
| Rotation speed | 100 rpm | 85.58±0.10^c^ | 93.70±0.01^c^ | 73.64±0.02^c^ |
|  | 120 rpm | 90.32±0.05^b^ | 96.07±0.01^b^ | 77.95±0.02^b^ |
|  | 150 rpm | 99.42±0.02^a^ | 98.47±0.06^a^ | 89.68±0.27^a^ |
|  | 180 rpm | 99.42±0.02^a^ | 98.47±0.09^a^ | 89.69±0.38^a^ |
|  | 200 rpm | 99.43±0.02^a^ | 98.47±0.02^a^ | 89.71±0.24^a^ |
| Initial nitrogen concentration | 100 mg/L | 99.61±0.17^a^ | 98.07±0.37^a^ | 87.86±0.11^b^ |
|  | 200 mg/L | 99.61±0.12^a^ | 98.01±0.13^a^ | 87.72±0.28^b^ |
|  | 400 mg/L | 99.41±0.01^a^ | 98.47±0.01^a^ | 89.68±0.16^a^ |
|  | 600 mg/L | 88.32±0.11^b^ | 91.11±0.01^b^ | 85.99±0.06^c^ |
|  | 800 mg/L | 86.00±0.04^c^ | 90.51±0.14^b^ | 77.02±0.25^d^ |
