# Supplementary material for: Enhanced nitrogen removal via simultaneous nitrification and denitrification by a newly isolated strain Enterobacter cloacae GW6 from estuarine sediment
Source: PLoS One. 2026 May 15;21(5):e0349379. doi: 10.1371/journal.pone.0349379 (PMC13178893; doi:10.1371/journal.pone.0349379)
Supplement: S4 Figure — Markers: a, 10000 bp; b, 5000 bp; c, 3000 bp; d, 2000 bp; e, 1500 bp; f, 1000 bp; g, 750 bp; h, 500 bp; i, 250 bp; g, 100 bp. (DOCX) [file pone.0349379.s004.docx]

**S4 Figure.** The PCR amplification of *amoA*, *hao*, *napA*, *nirK* and *nosZ* functional genes from *Enterobacter cloacae* GW6. Markers: a, 10000 bp; b, 5000 bp; c, 3000 bp; d, 2000 bp; e, 1500 bp; f, 1000 bp; g, 750 bp; h, 500 bp; i, 250 bp; g, 100 bp.
