# Supplementary material for: Enhanced nitrogen removal via simultaneous nitrification and denitrification by a newly isolated strain Enterobacter cloacae GW6 from estuarine sediment
Source: PLoS One. 2026 May 15;21(5):e0349379. doi: 10.1371/journal.pone.0349379 (PMC13178893; doi:10.1371/journal.pone.0349379)
Supplement: S5 Figure — Arrows in purple color indicate the heterotrophic nitrification pathway, while arrows in orange color indicate the aerobic denitrification pathway. (DOCX) [file pone.0349379.s005.docx]

**S5 Figure.** Proposed pathway of heterotrophic nitrification and aerobic denitrification of *Enterobacter cloacae* GW6 based on functional gene analysis using PCR. Arrows in purple color indicate the heterotrophic nitrification pathway, while arrows in orange color indicate the aerobic denitrification pathway.
